# Correlations among quality of life, spinal mobility, and disease activity in early-treated axial spondyloarthritis: a single-center cross-sectional study

**DOI:** 10.1186/s41927-024-00426-2

**Published:** 2024-10-16

**Authors:** Tinh Khampaen, Thanuchporn Kafaksom, Nichapa Dechapaphapitak, Nattakirana Tongdee, Parawee Chevaisrakul

**Affiliations:** Division of Allergy, Immunology, and Rheumatology, Department of Medicine, Faculty of Medicine Ramathibodi Hospital, Mahidol University, 270 Rama VI Road, Thung Phayathai Subdistrict, Ratchathewi, Bangkok, 10400 Thailand

**Keywords:** Axial spondyloarthritis, Early-treated, Disease activity, Quality of life, Functional ability, Spinal mobility

## Abstract

**Background:**

Axial spondyloarthritis (axSpA) significantly impacts patients’ lives. The ASAS-OMERACT guideline was formulated for the multidimensional evaluation of axSpA patients, employing a specific set of tools. Given the pivotal role of patient perception, comprehensive correlation among these tools, especially concerning quality of life, may provide a clinically relevant perspective and enhance treatment efficacy in the early stages of the disease. This study aims to investigate the correlation among disease activity, functional ability, and quality of life in early-treated axSpA patients. In addition, the association between high disease activity and clinical characteristics was explored.

**Methods:**

This cross-sectional study was conducted in a tertiary hospital in Thailand. Patients diagnosed with axSpA according to ASAS classification criteria and receiving treatment from rheumatologists within three years of onset of symptoms were included. Clinical and laboratory data were retrieved from a hospital database. Disease activity was assessed using the Ankylosing Spondylitis Disease Activity Score with ESR or CRP (ASDAS-ESR/CRP) and Bath Ankylosing Spondylitis Disease Activity Index (BASDAI). Spinal mobility was measured using the Bath Ankylosing Spondylitis Metrology Index (BASMI), while quality of life and function were evaluated using the ASAS Health Index (ASAS-HI) and Bath Ankylosing Spondylitis Functional Index (BASFI), respectively. The correlation between these measurements was analyzed using the Pearson correlation coefficient (r). Additionally, factors associated with high disease activity (ASDAS/CRP > 2.1) were explored using multivariate regression analysis.

**Results:**

Sixty-six patients (41 males; mean age 49.3 ± 13.3 years) were enrolled between April to December 2022. Disease activity (ASDAS-CRP) was significantly inversely correlated with spinal mobility (BASMI), function (BASFI), and quality of life (ASAS-HI). High disease activity was associated with obesity (BMI ≥ 30 kg/m^2) and a longer duration of symptoms before treatment (≥ 2 years).

**Conclusion:**

In early-treated axSpA patients, ASDAS-CRP showed significant correlations with functional ability, quality of life, and spinal mobility. High disease activity was associated with obesity and a longer pre-treatment symptom duration in our study. Early treatment may enhance patients’ function, mobility, and quality of life, with weight reduction being possibly beneficial for obese axSpA patients.

**Clinical trial number:**

Not applicable.

**Supplementary Information:**

The online version contains supplementary material available at 10.1186/s41927-024-00426-2.

## Introduction

Axial spondyloarthritis (axSpA) classically includes ankylosing spondylitis (AS) and non-radiographic axial spondyloarthritis (nr-axSpA). However, psoriatic arthritis (PsA) is typically categorized as peripheral spondyloarthritis, which may present axial components and also fulfill criteria of axSpA (some may represent as axPsA) [1, 2]. The Ankylosing Spondylitis Disease Activity Score with CRP (ASDAS-CRP) has emerged as a preferred tool for disease assessment in current practice recommendations due to its longitudinal correlation with syndesmophyte formation, which influences spinal mobility [3–7].

Given the pivotal role of patient perception, the ASAS Health Index (ASAS-HI) is designed to gauge the quality of life (QoL) across various domains like pain, emotional well-being, sleep, and mobility [8]. However, the practical application of ASAS-HI in routine clinical practice faces challenges with its complexity and the wide-ranging aspects it encompasses [8]. Understanding the intricate relationships between disease activity, functional ability, and quality of life, particularly regarding ASAS-HI, may provide a clinically relevant perspective and enhance treatment efficacy [9–13].

Many modifiable factors might attenuate the efficacy of treatment in axSpA patients. For example, the correlations between spinal mobility and disease activity emphasize the role of treatment in the early stage of axSpA to prevent structural damage to the spine, influencing spinal mobility [14]. Obese axSpA patients exhibit higher disease activity, more functional impairment and are refractory to treatment [15]. Sleep disturbance is also reported as a predictor of poor functional outcomes in axSpA patients, emphasizing the need for comprehensive evaluation and intervention [16, 17].

This study endeavors to bridge knowledge gaps by investigating the connection among disease activity, functional ability and quality of life in early-treated axSpA patients. The results may offer practical insights for clinicians managing these patients, especially those at risk of unfavorable outcomes, to optimize holistic care.

## Methods

### Study design

This cross-sectional study was conducted from April to December 2022, took place at the Division of Allergy, Immunology, and Rheumatology, Department of Medicine, Faculty of Medicine Ramathibodi Hospital, Mahidol University. Written informed consent was obtained from all participants and the study received approval from the Human Research Ethics Committee, Faculty of Medicine Ramathibodi Hospital, Mahidol University (MURA2022/252) according to the Declaration of Helsinki, the Belmont Report. An institutional Review Board (IRB) reviewed the data safety in accordance with the personal data protection act (PDPA), study conduction, and scientific validity throughout the research to maintain its integrity.

### Participants

Eligible patients were ≥ 18 years of age and diagnosed with axial spondyloarthritis (axSpA) according to Assessment of Spondyloarthritis International Society (ASAS) classification criteria [18]. Patients must have been receiving treatment from rheumatologists at the Ramathibodi Hospital Rheumatology Clinic within three years of the onset of symptoms. Patients who were ineligible to obtain BASDAI, BASMI, BASFI, ASAS-HI, and ASDAS or those who lost follow-up of more than one year were excluded.

### Procedures

After obtaining written informed consent, demographic data comprising gender, age, disease duration, disease features, comorbidities, treatment, and laboratory tests were collected. The same investigator reviewed and examined all participants throughout the study period. Demographic data and laboratory results were retrieved from a hospital database. A set of questionnaires was used to collect disease activity (ASDAS-ESR/CRP, BASDAI), function, and quality of life (BASFI, and ASAS-HI) [3, 8, 19, 20]. To assess spinal mobility using BASMI which measures range of motion through the tragus-to-wall test, cervical rotation test, modified lumbar Schober’s test, lumbar side flexion test, and intermalleolar distance were recorded and reported as a 2-point definition and 10-step definition composite index, BASMI2 and BASMI10, respectively [21]. These questionnaires have been validated in the Thai version [22].

### Statistical analysis

To evaluate the influence of modifiable factors such as obesity, sleep disturbance, and mood disorder on disease activity in axSpA patients, stratified by disease activity, ASDAS-CRP ≥.

2.1, we collected data of body mass index (BMI), Mood disorder was defined using Hospital Anxiety and Depression Scale (Thai HADS) [23], while sleep disturbance was determined by the answer of question number 16 in the ASAS-HI questionnaire (yes or no).

Demographic data were analyzed using descriptive statistics. Pearson correlation coefficients (r) were employed to assess the relationships between disease activity, spinal mobility, function, and quality of life. Multivariate regression analysis was conducted to identify factors associated with disease activity. For comparisons between groups, p-values were obtained using independent samples t-tests or Mann-Whitney U tests for continuous variables, and Chi-square tests or Fisher’s exact tests for categorical variables. For comparisons involving more than two groups, ANOVA tests were used. All statistical analyses were performed using Stata, with a significance level set at a two-tailed p-value < 0.05.

The sample size was calculated based on the correlation coefficient between disease activity and quality of life measures, using a p-value of 0.05 and a power of 0.80. This resulted in a required sample size of 29 patients to detect a moderate correlation (*r* = 0.5) among disease activity, function, quality of life, and spinal mobility measures.

## Results

The study included 66 patients identified as having early-treated axSpA. The majority of these patients were male (61.2%). Among the participants, 48 (72.3%) were classified as having radiographic axSpA according to the Modified New York criteria for ankylosing spondylitis [24]. Psoriasis was present in 18.2% of the patients. The HLA-B27 antigen was detected in 60% of the participants, with a higher prevalence in those with ankylosing spondylitis (90%) and radiographic axSpA (81.2%). Peripheral joint involvement was present in 37.9% of the patients. 9 (13.6%) patients were diagnosed with psoriatic arthritis (PsA) with axial radiographic changes in this study group before enrollment. Detailed clinical and laboratory data are presented in Table 1.

**Table 1:** Demographic data

	Total (n = 66)	ASDAS-CRP < 2.1 (n = 37)	ASDAS-CRP ≥ 2.1 (n = 29)	P-value
Age, years, mean (SD)	49.3 (13.5)	49.08 (14.01)	49.65 (13.13)	0.868
Male sex, n (%)	41 (62.1)	22 (59.46)	19 (65.52)	0.615
BMI, mean (SD)	25.9 (6.2)	24.7 (3.57)	27.5 (8.21)	0.060
- BMI < 30, n (%) *	54 (81.8)	34 (91.9)	20 (66.7)	0.017
- BMI ≥ 30, n (%)	12 (18.2)	3 (8.1)	9 (31.3)	
HLA-B27 positivity, n (%)	44	23 (62.16)	21 (72.41)	0.381
Radiographic change, n (%)	48	25 (67.57)	23 (79.31)	0.288
Smoking, n (%)	10	4 (10.8)	6 (20.69)	0.315
Previous duration of diagnosis before enrollment, years, median (IQR)	8 (4–12)	8 (3–10)	9 (4–15)	0.164
Duration of symptoms prior to treatment, years, mean (SD)*	1.2 (1.2)	0.9 (1.1)	1.6 (1.3)	0.034
- ≤ 2 years, n (%)	43 (65.2)	29 (78.4)	14 (48.3)	0.011
- > 2 years, n (%)	23 (34.8)	8 (21.6)	15 (51.7)	
Peripheral joint involvement, n (%)	25 (37.9)	12 (32.4)	13 (44.8)	0.303
- TJC-28, mean (SD)	0.4 (1.8)	0.1 (0.3)	0.8 (2.7)	0.020
- SJC-28, mean (SD)	0.3 (1.6)	0	0.7 (2.5)	0.009
Enthesitis, n (%)	36 (54.5)	19 (51.4)	17 (58.6)	0.556
Previous uveitis, n (%)	25 (37.9)	13 (35.1)	12 (41.4)	0.604
Syndesmophyte presence, n (%)*	30 (45.5)	11 (29.7)	19 (65.5)	0.004
The highest grade of sacroiliitis, median (IQR)	3 (2–4)	3 (2–3)	3 (2–4)	0.155
Psoriasis, n (%)	12 (18.2)	8 (21.6)	4 (13.8)	0.413
Nail abnormalities, n (%)	11 (16.7)	7 (19.0)	4 (13.8)	0.743
Inflammatory bowel disease (%)	1 (1.5)	0	1 (3.45)	0.439
WBC (x 10^3 cumm), mean (SD)*	7.0 (2.1)	6.1 (1.6)	8.2 (2.1)	< 0.001
Lymphocyte count (x 10^3 cumm), mean (SD)	2.0 (0.8)	2.0 (0.9)	2.1 (0.9)	0.661
Hemoglobin (g/dL), mean (SD)	12.7 (1.9)	12.9 (1.8)	12.4 (2.0)	0.274
Platelet count (x 10^3 cumm), mean (SD)	280 (73)	268 (75)	294 (70)	0.164
ESR (mm/Hg), median (IQR)*	28 (15–49)	23 (7–38)	41 (24–67)	0.002
CRP (mg/dL), median (IQR)*	2.5 (0-14.1)	1.5 (0-2.4)	16.2 (7.2–28.9)	< 0.001
Concurrent Medication usage, n (%)				
- NSAIDS	44 (66.7)	20 (54.1)	22 (75.9)	0.068
- MTX*	34 (51.5)	15 (40.5)	19 (65.5)	0.044
- SSZ	56 (84.8)	30 (81.1)	26 (84.9)	0.493
- LEF	16 (24.2)	6 (16.2)	10 (34.5)	0.086
- Glucocorticoid*	7 (10.6)	1 (2.7)	6 (20.7)	0.038
Biologic usage, n (%)	16 (24.2)	12 (32.4)	4 (13.79)	0.079
- Anti TNF	11 (16.7)	9 (24.3)	2 (6.9)	0.095
- Secukinumab	5 (7.6)	3 (8.1)	2 (6.9)	1.000

*P-value < 0.05

### The correlation between disease activity, spinal mobility and quality of life/function

Multiple assessment tools provide a multi-dimensional perspective on the disease. However, routine clinical practice may face challenges due to the complexity of these tools, especially in evaluating the quality of life.

Given the pivotal role of patient perception, a comprehensive correlation between disease activity (ASDAS-ESR/CRP), mobility (BASMI), and, particularly, quality of life and function (ASAS-HI, BASFI) was performed to offer a clinically relevant perspective and enhance the efficacy of treatment in the early stage of the disease (refer to Table 2).

In early-treated axSpA, the study revealed a robust correlation between tools evaluating disease activity, specifically ASDAS-CRP and BASDAI (rho = 0.7260, *p* < 0.0001). Notably, moderate correlations emerged among tools assessing disease activity (ASDAS-CRP), function (BASFI), and quality of life (ASAS-HI) (rho = 0.5443 and 0.5434, *p* < 0.0001 for both). Nonetheless, the correlation between disease activity (ASDAS-CRP) and spinal mobility (BASMI) was low (rho = 0.3338, *p* = 0.0062). Additionally, a strong correlation was found between tools determining mobility (BASMI 2-point and BASMI 10-point systems) (rho = 0.8838, *p* < 0.0001). Unfortunately, ASDAS-ESR showed moderate to low correlations with other tools.

**Fig. 1:**
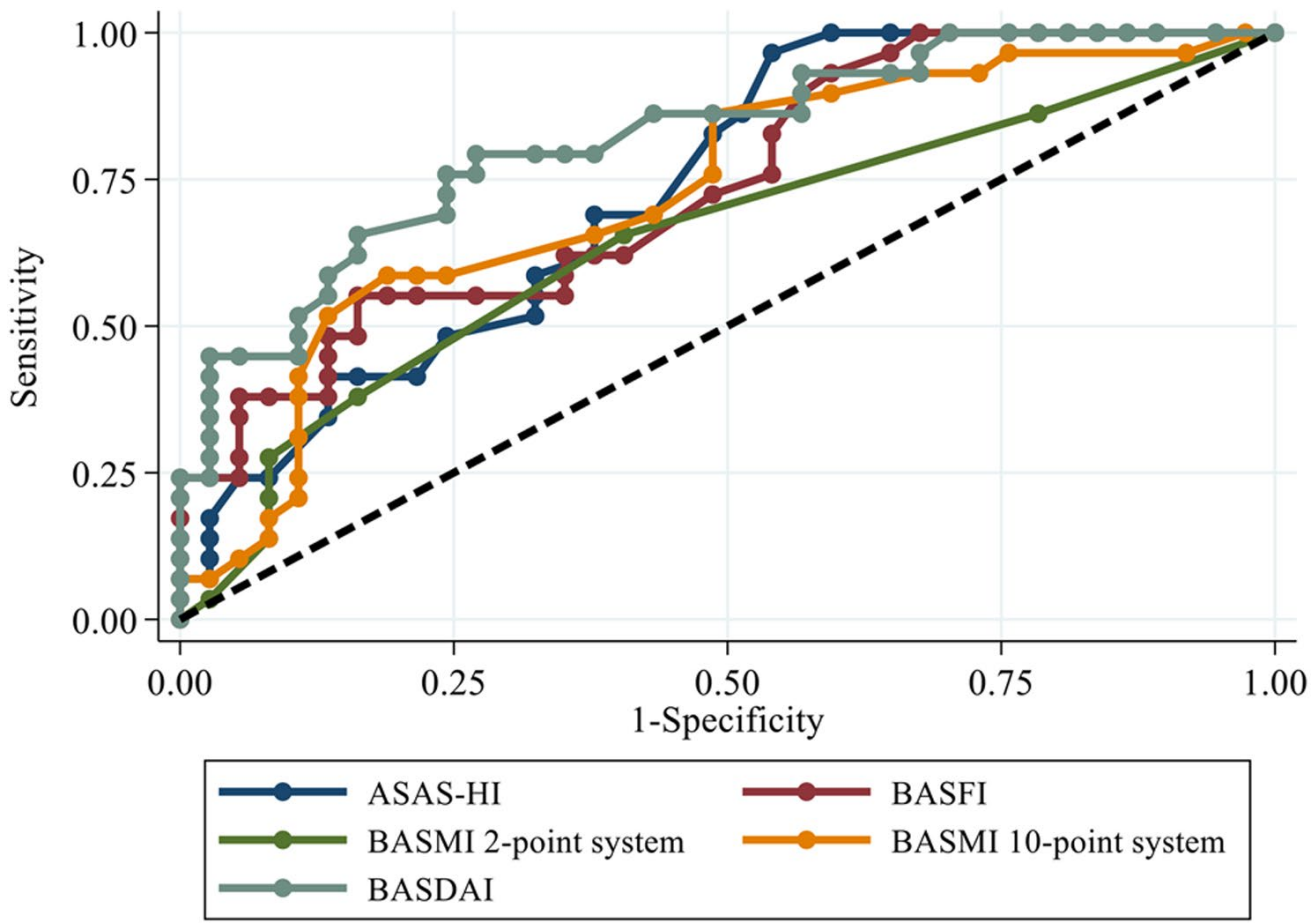
ROC Curve between high disease activity (ASDAS-CRP ≥ 2.1) and other clinical assessment

When comparing tool performance to assess disease activity defined by using a cut-off ASDAS-CRP > 2.1 (balancing between sensitivity and specificity) among tools evaluating disease activity, mobility, and quality of life, we used ROC curve analysis (refer to Fig. 1). As expected, ASDAS-ESR exhibited the highest performance (AUC 0.8295), followed by BASFI, ASAS-HI, and BASMI10. BASMI2 had the lowest performance in predicting high disease activity (AUC 0.6459).

**Table 2:** Correlation matrix (Spearman’s rho) of the ASDAS-ESR/CRP versus other disease activity score without laboratory tests (BASDAI), function (BASFI), quality of life (ASAS-HI), and spinal mobility measurements (BASMI)

	BASDAI	BASFI	ASAS-HI	BASMI 2-point system	BASMI 10-point system
ASDAS-CRP	0.7260 <0.0001	0.5443 <0.0001	0.5434 <0.0001	0.1918 0.1230	0.3338 0.0062
ASDAS-ESR	0.7040 <0.0001	0.4968 <0.0001	0.5640 <0.0001	0.1360 <0.0001	0.2254 0.0689
BASDAI		0.5776 <0.0001	0.6558 <0.0001	-0.0896 0.4744	0.0261 0.8355
BASFI			0.7435 <0.0001	-0.0551 0.6602	0.2693 0.0288
ASAS-HI				-0.0551 0.6602	0.0698 0.5774
BASMI 2-point system					0.8838 <0.0001

## Factors predicting high disease activity in early-treated axSpA

To optimize holistic care in the early stages of disease, it is crucial to identify individuals at risk of unfavorable outcomes. Although the sample size of this study was not specifically estimated for this purpose, it is worthwhile to analyze whether there is an association between modifiable factors and high disease activity (ASDAS > 2.1).

We categorized patients into groups of high and low disease activity based on ASDAS-CRP scores at a threshold of 2.1 and observed that both groups exhibited similar age ranges and gender distributions. No significant differences were noted in HLA-B27 status, radiographic damage, smoking habits, peripheral joint involvement, enthesitis, and previous uveitis (Table 1). There is no difference in the use of conventional DMARDs between the two groups. Notably, only 24.2% of patients had received biologics, and there were no significant differences in disease activity between biologics users and non-users. However, we observed a trend towards a higher proportion of biologics users achieving low disease activity.

In contrast, several notable differences were observed between the groups. The high disease activity group had a higher mean body mass index (BMI) of 27.5 compared to 24.7 in the low disease activity group, as well as a significantly greater prevalence of obesity (31.3% vs. 8.1%). Longer disease duration appeared to correlate with higher disease activity, as the high disease activity group had a slightly longer median disease duration (9 years vs. 8 years) and a greater percentage of patients with disease onset exceeding 10 years (41.5% vs. 16.5%) (see Table 1). More specifically, the high disease activity group had a longer duration before starting treatment (1.6 years vs. 0.9 years).

We stratified patients into three groups based on the duration of time before treatment: less than 1 year, 1–2 years, and 2–3 years. There was no significant difference in the proportion of patients exhibiting high disease activity among the groups (27.3%, 41.7%, and 68.1%, respectively; *p* = 0.206). However, we did observe a trend indicating that shorter time to treatment was associated with lower disease activity. When further stratifying patients based on disease duration prior to treatment using a cutoff of 2 years, we found that a higher percentage of patients with a longer time to treatment exhibited high disease activity (51.7% vs. 21.6%, *p* = 0.011). Similarly, when using a cutoff of 1 year, we also noted that a greater percentage of patients with a longer time to treatment exhibited high disease activity (60.6% vs. 27.3%, *p* = 0.021).

Moreover, patients experiencing high disease activity also demonstrated elevated levels of sleep disturbance (62% vs. 35%, *p* = 0.030) and higher Hospital Anxiety and Depression Scale–Depression (HADS-D) scores, indicating increased symptoms of depression (*p* = 0.026). Additional data can be found in Supplementary Appendix Table 1. In multivariate analysis, it was found that obesity (OR 6.24, P-value 0.027) and longer disease duration before treatment (OR 7.33, P-value 0.003) were significantly associated with high disease activity (Table 3).

**Table 3:** Univariate analysis and multivariate analysis for high disease activity (ASDAS-CRP $\:\ge\:$ 2.1)

Risk factor	Univariate analysis		Multivariate analysis	
	Odd ratios	P-value	Odd ratios	P-value
BMI ≥ 30	5.09 (1.23–21.07)	0.024	6.24 (1.23–31.63)	0.027
HADS-D 8–10	3.67 (0.82–16.43)	0.090	3.55 (0.63–19.86)	0.149
HADS-D ≥ 11	9.17 (0.99–84.61)	0.051	8.72 (0.79–95.73)	0.077
Sleep disturbance	3.02 (1.10–8.28)	0.032	3.18 (0.88–11.51)	0.077
Duration of symptoms prior to treatment > 2 years	3.88 (1.33–11.31)	0.013	7.33 (1.93–27.91)	0.003
Syndesmophyte presence	4.49 (1.58–12.71)	0.003		
WBC count	1.000624 (1.000281- 1.000967)	< 0.001		

## Discussion

Axial spondyloarthritis (axSpA) significantly impacts multiple dimensions of patient health, including disease activity, spinal mobility, and quality of life, with associated costs often underestimated. Establishing correlations among assessment tools—particularly those measuring quality of life—can enhance the efficacy of early treatment strategies.

In our study of early-treated axSpA patients, we found strong correlations among disease activity, spinal mobility, and quality of life, aligning with previous studies on long-standing disease [9, 25]. In clinical practice, simpler assessment tools are often preferred due to time limitations. The Ankylosing Spondylitis Disease Activity Score (ASDAS-CRP) well reflected for evaluating quality of life and function; however, the modest correlation observed between disease activity and spinal mobility indicates that insufficient treatment may not effectively prevent structural damage. Additionally, targeted therapies that have been shown to delay radiographic progression [26–29] were used by fewer than one-third of our participants during a time when biologics were not widely reimbursed nationally. This limitation restricts our ability to compare disease activity between biologic and non-biologic users due to the small sample size.

The context of our findings helps explain the high usage of conventional DMARDs, as many patients could not afford biologic treatments. Notably, our data revealed that a significant proportion of non-biologic users also achieved low disease activity. Previous studies have reported that a combination of sulfasalazine and methotrexate can reduce disease activity and the need for biologics [30]. Patients with axSpA may experience a partial response to conventional DMARDs, as indicated by the predominant drug use in our study, particularly in settings with limited resources. Nonetheless, we observed similar treatment responses when using either 1 year or 2 years as the cutoff for time to treatment. Our results illustrate the effects of time to treatment based on these cutoffs.

We believe that the lack of significant differences when comparing the three groups (< 1 year, 1–2 years, and 2–3 years) can be attributed to the minimal difference between the < 1 year and 1–2 year groups. In contrast, those treated later than 2 years demonstrated a significant lower response. A prior study defined “2 years” as the threshold for early axSpA [31], which supports the relevance of this cutoff in our findings. These data may reinforce the concept of a “window of opportunity” for treatment in early axial spondyloarthritis.

Additionally, our multivariate regression analysis indicated that a longer disease duration before treatment is linked to an increased risk of unfavorable outcomes, confirming our earlier findings. We also found obesity to be associated with higher disease activity. Previous reports have identified depression and sleep disturbances as factors correlated with increased disease activity; however, these variables could not be adequately explored within the limited sample size of our study. This underscores the vital role of primary healthcare in the early detection of disease, particularly for individuals presenting with chronic inflammatory back pain. It also highlights the importance of a multidisciplinary approach in treating axSpA, especially in patients with obesity.

Notably, 18.2% of our participants had psoriasis, but only 13% were previously diagnosed with psoriatic arthritis (PsA) before being enrolled in our study. We classified individuals based on clinical back pain, enrolling those who met the Assessment of Spondyloarthritis International Society (ASAS) classification criteria. This group includes both ankylosing spondylitis (AS/axSpA) associated with psoriasis and axial psoriatic arthritis (axPsA). Given the clinical overlap we assumed between these entities, we chose not to differentiate between them. Our primary focus remained on disease activity and the patient assessment of “axial symptoms.” However, a limitation of our study is the potential existence of distinct clinical features between these two entities, even though we currently apply the same treatment algorithm for both axSpA and axPsA.

Our study underscores the significance of syndesmophytes as a poor prognostic indicator in early-treated axSpA patients [32, 33]. The presence of syndesmophytes despite early treatment suggests worse outcomes, necessitating closer monitoring. Additionally, we found a high white blood cell count indicative of heightened disease activity [34], though no significant associations were detected with platelet or lymphocyte counts, likely due to our limited participant pool.

Our study’s strengths lie in the focused inclusion of early-treated axSpA patients, a critical treatment period often overlooked. The comprehensive range of measures provides a nuanced understanding of the disease’s impact, with the analysis of factors associated with higher disease activity offering valuable insights. Future research endeavors should consider these findings, addressing the highlighted limitation and further exploring the unique characteristics of early-treated axSpA patients for a more comprehensive understanding of their outcomes.

Nevertheless, the study had limitations beyond the sample size, including participant enrollment in a single tertiary hospital setting, potentially influencing selection bias and the generalizability of the results. Additionally, the study did not investigate the impact of certain comorbidities on the disease outcome, such as degenerative spine diseases and fibromyalgia [35].

## Conclusion

ASDAS-CRP, a currently recommended disease activity measure for axSpA, demonstrated significantly inverse correlation with poorer spinal mobility and quality of life, in early-treated axSpA patients. Our study found associations between obesity, long disease duration before treatment and disease activity. Consequently, early treatment and a multidisciplinary team, including primary healthcare, may enhance treatment outcomes.

## Electronic supplementary material

Below is the link to the electronic supplementary material.

Supplementary Material 1

## Data Availability

The datasets generated and/or analysed during the current study are available in the figshare repository, 10.6084/m9.figshare.25440484[36].

## Abbreviations

ASDAS-ESR/CRP: Ankylosing Spondylitis Disease Activity Score with ESR or CRP
axSpA: axial spondyloarthritis
BASDAI: Bath Ankylosing Spondylitis Disease Activity Index
BASFI: Bath Ankylosing Spondylitis Functional Index
BASMI: Bath Ankylosing Spondylitis Metrology Index
CASPAR: Classification for Psoriatic Arthritis
CRP: C-reactive protein
DMARDs: Disease-Modifying Anti-Rheumatic Drugs
HADS: Hospital Anxiety and Depression Scale
HLA: Human Leucocyte Antigen
NSAIDs: Nonsteroidal Anti-inflammatory Drugs
PsA: psoriatic arthritis
ROC: Receiver Operating Characteristic
SD: standard deviation
SJC-28: Swollen Joint Count of 28 joints
TJC-28: Tender Joint Count of 28 joints
TNF: Tumor Necrosis Factor
WBC: White Blood Cell

## References

[1] Navarro-Compán V, Sepriano A, El-Zorkany B, van der Heijde D. Axial spondyloarthritis. Ann Rheum Dis. 2021;80(12):1511–21. 34615639 10.1136/annrheumdis-2021-221035

[2] Sieper J, Poddubnyy D. Axial spondyloarthritis. Lancet. 2017;390(10089):73–84. 28110981 10.1016/S0140-6736(16)31591-4

[3] van der Heijde D, Lie E, Kvien TK, Sieper J, Van den Bosch F, Listing J, et al. ASDAS, a highly discriminatory ASAS-endorsed disease activity score in patients with ankylosing spondylitis. Ann Rheum Dis. 2009;68(12):1811–8. 19060001 10.1136/ard.2008.100826

[4] Lukas C, Landewé R, Sieper J, Dougados M, Davis J, Braun J, et al. Development of an ASAS-endorsed disease activity score (ASDAS) in patients with ankylosing spondylitis. Ann Rheum Dis. 2009;68(1):18–24. 18625618 10.1136/ard.2008.094870

[5] Machado P, Landewé R, Lie E, Kvien TK, Braun J, Baker D, et al. Ankylosing Spondylitis Disease Activity score (ASDAS): defining cut-off values for disease activity states and improvement scores. Ann Rheum Dis. 2011;70(1):47–53. 21068095 10.1136/ard.2010.138594

[6] Ramiro S, van der Heijde D, van Tubergen A, Stolwijk C, Dougados M, van den Bosch F, et al. Higher disease activity leads to more structural damage in the spine in ankylosing spondylitis: 12-year longitudinal data from the OASIS cohort. Ann Rheum Dis. 2014;73(8):1455–61. 24812292 10.1136/annrheumdis-2014-205178

[7] Poddubnyy D, Protopopov M, Haibel H, Braun J, Rudwaleit M, Sieper J. High disease activity according to the Ankylosing spondylitis Disease activity score is associated with accelerated radiographic spinal progression in patients with early axial spondyloarthritis: results from the GErman SPondyloarthritis Inception Cohort. Ann Rheum Dis. 2016;75(12):2114–8. 27125522 10.1136/annrheumdis-2016-209209

[8] Kiltz U, van der Heijde D, Boonen A, Cieza A, Stucki G, Khan MA, et al. Development of a health index in patients with ankylosing spondylitis (ASAS HI): final result of a global initiative based on the ICF guided by ASAS. Ann Rheum Dis. 2015;74(5):830–5. 24399232 10.1136/annrheumdis-2013-203967 PMC4511705

[9] Di Carlo M, Lato V, Carotti M, Salaffi F. Clinimetric properties of the ASAS health index in a cohort of Italian patients with axial spondyloarthritis. Health Qual Life Outcomes. 2016;14:78. 27188166 10.1186/s12955-016-0463-1 PMC4869300

[10] Hamdi W, Ghannouchi MM, Zouch I, Kchir MM. AB0915 ankylosing spondylitis: the correlation between mobility limitation score (BASMI) and a new disease activity index (ASDAS). Ann Rheum Dis. 2013;71(Suppl 3):691.

[11] Popescu C, Trandafir M, Bădică A, Morar F, Predeţeanu D. Ankylosing spondylitis functional and activity indices in clinical practice. J Med Life. 2014;7(1):78–83. 24653763 PMC3956102

[12] Alonso Castro S, Pardo Campo E, Charca Benavente LC, Pino Martínez M, Fernández S, Arboleya Rodríguez LM, AB0717 THE ASAS-HEALTH INDEX MAY BE USEFUL TO IDENTIFY DISEASE ACTIVITY STATES IN PATIENTS WITH SPONDYLOARTHRITIS, et al. Ann Rheum Dis. 2020;79(Suppl 1):1654.

[13] Schneeberger EE, Citera G, de Leon DP, Szumski AE, Kwok K, Cutri M, et al. Simplified Ankylosing Spondylitis Disease Activity score (SASDAS) Versus ASDAS: a Post Hoc Analysis of a Randomized Controlled Trial. J Rhuematol. 2022;49(10):1100. 10.3899/jrheum.211075 35840157

[14] Chung HY, Chan SCW, Lee KH, Tsang HHL, Ng LL, Lau CS. Both ASDAS and ADC are associated with spinal mobility in active axial spondyloarthritis: a comparison between early and later disease. Int J Rheum Dis. 2022;25(3):317–26. 35019230 10.1111/1756-185X.14278

[15] Maas F, Arends S, van der Veer E, Wink F, Efde M, Bootsma H, et al. Obesity is common in Axial Spondyloarthritis and is Associated with Poor Clinical Outcome. J Rhuematol. 2016;43(2):383–7. 10.3899/jrheum.150648 26669924

[16] Deodhar A, Gensler LS, Magrey M, Walsh JA, Winseck A, Grant D, et al. Assessing physical activity and sleep in Axial Spondyloarthritis: measuring the gap. Rheumatol Ther. 2019;6(4):487–501. 31673975 10.1007/s40744-019-00176-5 PMC6858410

[17] Macfarlane GJ, Barnish MS, Jones EA, Kay L, Keat A, Meldrum KT, et al. The British Society for Rheumatology Biologics Registers in Ankylosing Spondylitis (BSRBR-AS) study: protocol for a prospective cohort study of the long-term safety and quality of life outcomes of biologic treatment. BMC Musculoskelet Disord. 2015;16(1):347. 26559487 10.1186/s12891-015-0805-x PMC4642769

[18] Rudwaleit M, Landewé R, van der Heijde D, Listing J, Brandt J, Braun J, et al. The development of Assessment of SpondyloArthritis international society classification criteria for axial spondyloarthritis (part I): classification of paper patients by expert opinion including uncertainty appraisal. Ann Rheum Dis. 2009;68(6):770–6. 19297345 10.1136/ard.2009.108217

[19] Calin A, Garrett S, Whitelock H, Kennedy LG, O’Hea J, Mallorie P, et al. A new approach to defining functional ability in ankylosing spondylitis: the development of the bath ankylosing Spondylitis Functional Index. J Rheumatol. 1994;21(12):2281–5. 7699629

[20] Garrett S, Jenkinson T, Kennedy LG, Whitelock H, Gaisford P, Calin A. A new approach to defining disease status in ankylosing spondylitis: the bath ankylosing Spondylitis Disease Activity Index. J Rheumatol. 1994;21(12):2286–91. 7699630

[21] Jones SD, Porter J, Garrett SL, Kennedy LG, Whitelock H, Calin A. A new scoring system for the bath ankylosing Spondylitis Metrology Index (BASMI). J Rheumatol. 1995;22(8):1609. 7473496

[22] Kittiyanpanya C, Chaiamnuay S, Asavatanabodee P, Narongroeknawin P. Reliability and validity of the Thai version of bath ankylosing spondylitis indices. J Med Assoc Thai. 2014;97(4):381–5. 24964679

[23] Nilchaikovit T, Lotrakul M, Phisansuthideth U. Development of Thai version of hospital anxiety and depression scale in cancer patients. J Psychiatr Assoc Thai. 1996;41:18–30.

[24] van der Linden S, Valkenburg HA, Cats A. Evaluation of diagnostic criteria for ankylosing spondylitis. A proposal for modification of the New York criteria. Arthritis Rheum. 1984;27(4):361–8. 6231933 10.1002/art.1780270401

[25] Nas K, ÇEvİK R, SaraÇ AJ, GÜR A, Bozkurt M. Relationship between clinical findings, quality of life and functional disability related to Disease Activity in patients with Ankylosing Spondylitis. Turkish J Rheumatology()Archives Rheumatol. 2011;26(1):29–37.

[26] Sieper J, van der Heijde D, Dougados M, Mease PJ, Maksymowych WP, Brown MA, et al. Efficacy and safety of adalimumab in patients with non-radiographic axial spondyloarthritis: results of a randomised placebo-controlled trial (ABILITY-1). Ann Rheum Dis. 2013;72(6):815–22. 22772328 10.1136/annrheumdis-2012-201766 PMC3664374

[27] Baraliakos X, Østergaard M, Gensler LS, Poddubnyy D, Lee EY, Kiltz U, et al. Comparison of the effects of Secukinumab and Adalimumab Biosimilar on Radiographic Progression in patients with Ankylosing spondylitis: design of a Randomized, Phase IIIb Study (SURPASS). Clin Drug Investig. 2020;40(3):269–78. 31983056 10.1007/s40261-020-00886-7

[28] Baraliakos X, Haibel H, Listing J, Sieper J, Braun J. Continuous long-term anti-TNF therapy does not lead to an increase in the rate of new bone formation over 8 years in patients with ankylosing spondylitis. Ann Rheum Dis. 2014;73(4):710–5. 23505240 10.1136/annrheumdis-2012-202698

[29] Baraliakos X, Listing J, Rudwaleit M, Haibel H, Brandt J, Sieper J, et al. Progression of radiographic damage in patients with ankylosing spondylitis: defining the central role of syndesmophytes. Ann Rheum Dis. 2007;66(7):910–5. 17329306 10.1136/ard.2006.066415 PMC1955120

[30] Ganapati A, Gowri M, Antonisamy B, Danda D. Combination of methotrexate and sulfasalazine is an efficacious option for axial spondyloarthritis in a resource-limited, real-world clinical setting: a prospective cohort study. Clin Rheumatol. 2021;40(5):1871–9. 33058032 10.1007/s10067-020-05433-5

[31] Ciurea A, Götschi A, Bräm R, Bürki K, Exer P, Andor M et al. Early axial spondyloarthritis according to the ASAS consensus definition: characterisation of patients and effectiveness of a first TNF inhibitor in a large observational registry. RMD Open. 2023;9(4). 10.1136/rmdopen-2023-003455 PMC10693870 38053462

[32] Baraliakos X, Listing J, Rudwaleit M, Brandt J, Sieper J, Braun J. Radiographic progression in patients with ankylosing spondylitis after 2 years of treatment with the tumour necrosis factor alpha antibody infliximab. Ann Rheum Dis. 2005;64(10):1462–6. 15778240 10.1136/ard.2004.033472 PMC1755223

[33] Baraliakos X, Listing J, von der Recke A, Braun J. The natural course of radiographic progression in ankylosing spondylitis: differences between genders and appearance of characteristic radiographic features. Curr Rheumatol Rep. 2011;13(5):383–7. 21706179 10.1007/s11926-011-0192-8

[34] Sen R, Kim E, Napier RJ, Cheng E, Fernandez A, Manning ES, et al. Neutrophil-to-lymphocyte ratio and platelet-to-lymphocyte ratio as biomarkers in Axial Spondyloarthritis: Observational studies from the program to Understand the Longterm outcomes in Spondyloarthritis Registry. Arthritis Rheumatol. 2023;75(2):232–41. 36053919 10.1002/art.42333 PMC9892177

[35] Jones GT, Mallawaarachchi B, Shim J, Lock J, Macfarlane GJ. The prevalence of fibromyalgia in axial spondyloarthritis. Rheumatol Int. 2020;40(10):1581–91. 32556474 10.1007/s00296-020-04621-5 PMC7452944

[36] Khampaen T. AxSpA Dataset. figshare; 2024.

