# Correction: Correlations among quality of life, spinal mobility, and disease activity in early-treated axial spondyloarthritis: a single-center cross-sectional study

**DOI:** 10.1186/s41927-026-00644-w

**Published:** 2026-04-13

**Authors:** Tinh Khampaen, Thanuchporn Kafaksom, Nichapa Dechapaphapitak, Nattakirana Tongdee, Parawee Chevaisrakul

**Affiliations:** Division of Allergy, Immunology, and Rheumatology, Department of Medicine, Faculty of Medicine Ramathibodi Hospital, Mahidol University, 270 Rama VI Road, Thung Phayathai Subdistrict, Ratchathewi, Bangkok, 10400 Thailand

**Correction: BMC Rheumatol (2024) 8:54**

10.1186/s41927-024-00426-2

Following publication of the original article [1], misalignments were found in Tables 1 and 3, therefore they have been updated.

Table 1 has been corrected from:

**Table 1:**
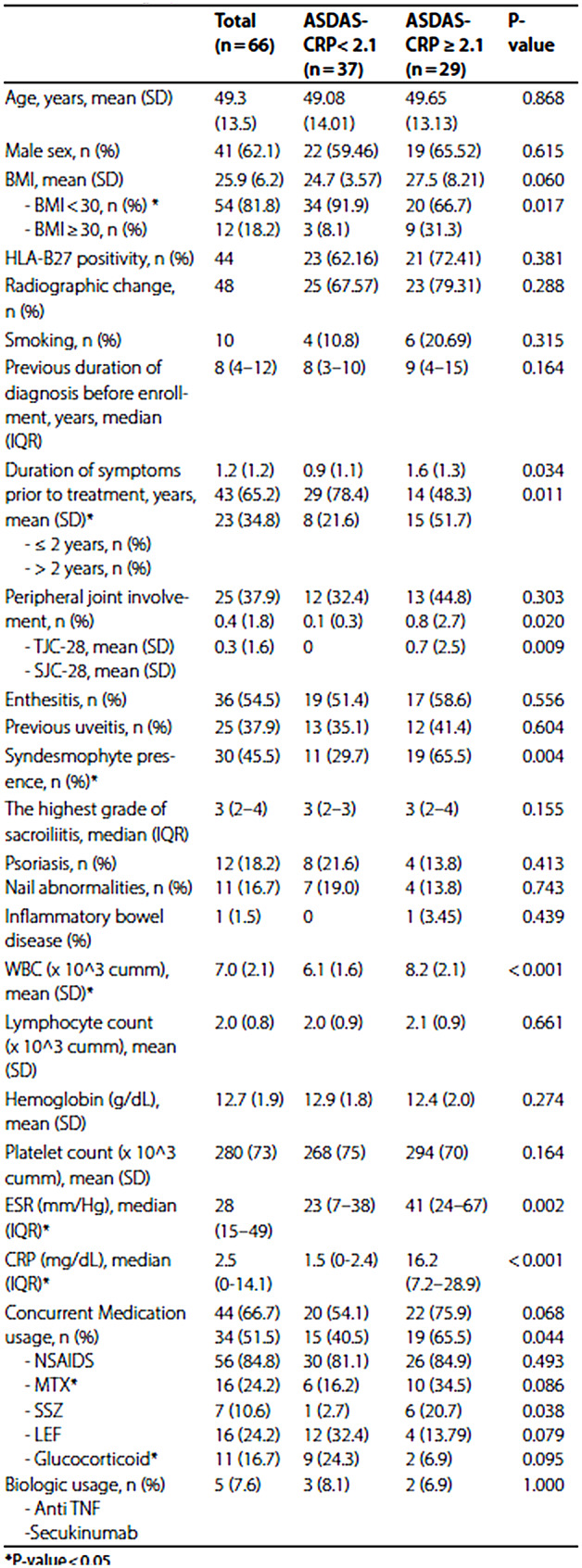
Demographic data

To:

**Table 1:** Demographic data

	Total (*n* = 66)	ASDAS-CRP < 2.1 (*n* = 37)	ASDAS-CRP ≥ 2.1 (*n* = 29)	*P*-value
Age, years, mean (SD)	49.3 (13.5)	49.08 (14.01)	49.65 (13.13)	0.868
Male sex, n (%)	41 (62.1)	22 (59.46)	19 (65.52)	0.615
BMI, mean (SD) - BMI < 30, n (%) * - BMI ≥ 30, n (%)	25.9 (6.2) 54 (81.8) 12 (18.2)	24.7 (3.57) 34 (91.9) 3 (8.1)	27.5 (8.21) 20 (66.7) 9 (31.3)	0.060 0.017
HLA-B27 positivity, n (%)	44	23 (62.16)	21 (72.41)	0.381
Radiographic change, n (%)	48	25 (67.57)	23 (79.31)	0.288
Smoking, n (%)	10	4 (10.8)	6 (20.69)	0.315
Previous duration of diagnosis before enrollment, years, median (IQR)	8 (4–12)	8 (3–10)	9 (4–15)	0.164
Duration of symptoms prior to treatment, years, mean (SD)* - ≤ 2 years, n (%) - > 2 years, n (%)	1.2 (1.2) 43 (65.2) 23 (34.8)	0.9 (1.1) 29 (78.4) 8 (21.6)	1.6 (1.3) 14 (48.3) 15 (51.7)	0.034 0.011
Peripheral joint involvement, n (%) - TJC-28, mean (SD) - SJC-28, mean (SD)	25 (37.9) 0.4 (1.8) 0.3 (1.6)	12 (32.4) 0.1 (0.3) 0	13 (44.8) 0.8 (2.7) 0.7 (2.5)	0.303 0.020 0.009
Enthesitis, n (%)	36 (54.5)	19 (51.4)	17 (58.6)	0.556
Previous uveitis, n (%)	25 (37.9)	13 (35.1)	12 (41.4)	0.604
Syndesmophyte presence, n (%)*	30 (45.5)	11 (29.7)	19 (65.5)	0.004
The highest grade of sacroiliitis, median (IQR)	3 (2–4)	3 (2–3)	3 (2–4)	0.155
Psoriasis, n (%) Nail abnormalities, n (%)	12 (18.2) 11 (16.7)	8 (21.6) 7 (19.0)	4 (13.8) 4 (13.8)	0.413 0.743
Inflammatory bowel disease (%)	1 (1.5)	0	1 (3.45)	0.439
WBC (x 10^3 cumm), mean (SD)*	7.0 (2.1)	6.1 (1.6)	8.2 (2.1)	< 0.001
Lymphocyte count (x 10^3 cumm), mean (SD)	2.0 (0.8)	2.0 (0.9)	2.1 (0.9)	0.661
Hemoglobin (g/dL), mean (SD)	12.7 (1.9)	12.9 (1.8)	12.4 (2.0)	0.274
Platelet count (x 10^3 cumm), mean (SD)	280 (73)	268 (75)	294 (70)	0.164
ESR (mm/Hg), median (IQR)*	28 (15–49)	23 (7–38)	41 (24–67)	0.002
CRP (mg/dL), median (IQR)*	2.5 (0-14.1)	1.5 (0-2.4)	16.2 (7.2–28.9)	< 0.001
Concurrent Medication usage, n (%)				
- NSAIDS - MTX* - SSZ - LEF - Glucocorticoid* Biologic usage, n (%) - Anti TNF - Secukinumab	44 (66.7) 34 (51.5) 56 (84.8) 16 (24.2) 7 (10.6) 16 (24.2) 11 (16.7) 5 (7.6)	20 (54.1) 15 (40.5) 30 (81.1) 6 (16.2) 1 (2.7) 12 (32.4) 9 (24.3) 3 (8.1)	22 (75.9) 19 (65.5) 26 (84.9) 10 (34.5) 6 (20.7) 4 (13.79) 2 (6.9) 2 (6.9)	0.068 0.044 0.493 0.086 0.038 0.079 0.095 1.000

*P-value < 0.05

Table 3 has been updated from:

**Table 3:**
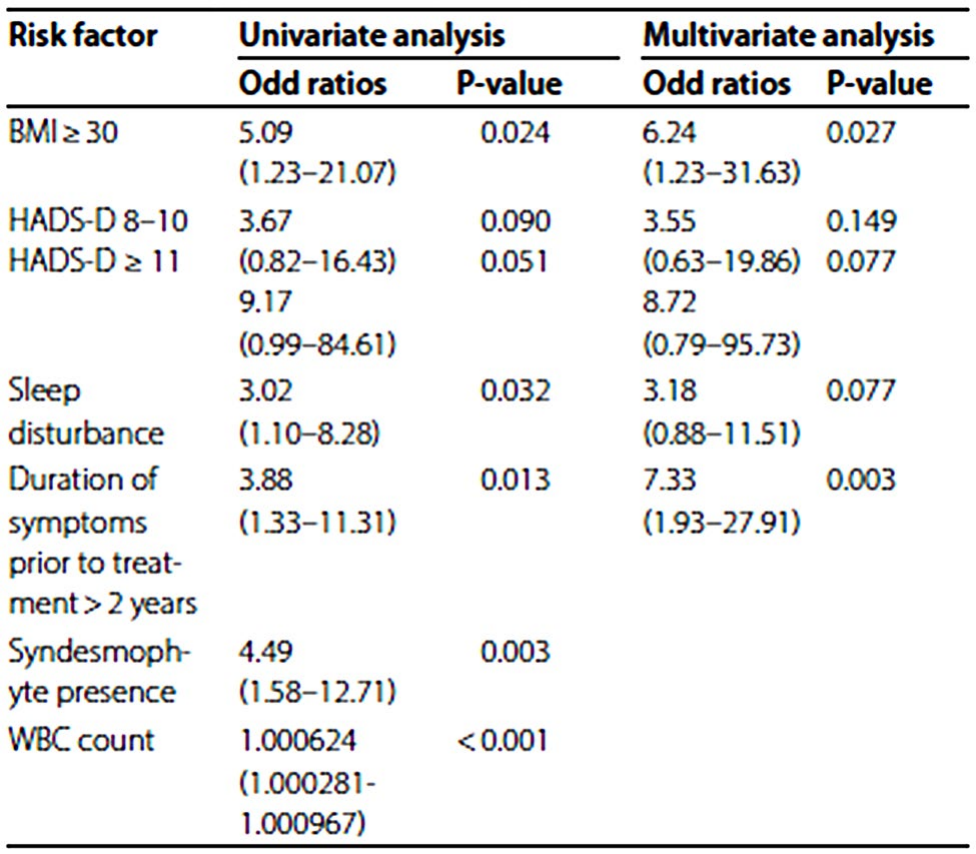
Univariate analysis and multivariate analysis for high disease activity (ASDAS-CRP $\:\ge\:$ 2.1)

To:

**Table 3:** Univariate analysis and multivariate analysis for high disease activity (ASDAS-CRP $\:\ge\:$ 2.1)

Risk factor	Univariate analysis		Multivariate analysis	
	Odd ratios	*P*-value	Odd ratios	*P*-value
BMI ≥ 30	5.09 (1.23–21.07)	0.024	6.24 (1.23–31.63)	0.027
HADS-D 8–10 HADS-D ≥ 11	3.67 (0.82–16.43) 9.17 (0.99–84.61)	0.090 0.051	3.55 (0.63–19.86) 8.72 (0.79–95.73)	0.149 0.077
Sleep disturbance	3.02 (1.10–8.28)	0.032	3.18 (0.88–11.51)	0.077
Duration of symptoms prior to treatment > 2 years	3.88 (1.33–11.31)	0.013	7.33 (1.93–27.91)	0.003
Syndesmophyte presence	4.49 (1.58–12.71)	0.003		
WBC count	1.000624 (1.000281- 1.000967)	< 0.001		

The original article [1] has been updated.
