# Supplementary material for: Correlations among quality of life, spinal mobility, and disease activity in early-treated axial spondyloarthritis: a single-center cross-sectional study
Source: BMC Rheumatol. 2024 Oct 16;8:54. doi: 10.1186/s41927-024-00426-2 (PMC11481462; doi:10.1186/s41927-024-00426-2)
Supplement: Supplementary file 1 — Supplementary Material 1 [file 41927_2024_426_MOESM1_ESM.docx]

**Supplementary Appendix**

**Supplementary Figure 1:** Diagnosis made with rheumatologists in early-treated patient axial spondyloarthritis (n=66)

**13 (19.7%)**

**40 (60.6%)**

**13 (19.7%)**

**Supplementary Table 1:** Area under ROC Curve between high disease activity (ASDAS-CRP ≥ 2.1) and other clinical assessment

|  | ROC Area | Standard Error | 95% Confidential Interval |
| --- | --- | --- | --- |
| BASDAI | 0.8201 | 0.0515 | 0.71914 ~ 0.92112 |
| ASAS-HI | 0.7330 | 0.0608 | 0.61385 ~ 0.85213 |
| BASFI | 0.7395 | 0.0607 | 0.62061 ~ 0.85842 |
| BASMI_2_ | 0.6459 | 0.0684 | 0.51185 ~ 0.77986 |
| BASMI_10_ | 0.7279 | 0.0634 | 0.60361 ~ 0.85213 |

**Supplementary Table 2: Comparison of assessment tools between low (ASDAS-CRP < 2.1) and high (ASDAS-CRP ≥ 2.1) disease activity**

|  | ASDAS-CRP < 2.1 (n = 37) | ASDAS-CRP >= 2.1 (n = 29) | P-value |
| --- | --- | --- | --- |
| BASFI, median (IQR) | 0.9 (0.2-1.6) | 2.3 (0.9-4.1) | 0.009 |
| BASDAI, median (IQR) | 2.4 (1.2-3.1) | 4.1 (3.3-6.1) | <0.001 |
| ASAS-HI, median (IQR) | 4.5 (3-7) | 7 (5-9) | 0.001 |
| Patient global assessment, mean (SD) | 2.4 (1.4) | 4.6 (2.2) | <0.001 |
| Physician global assessment, mean (SD) | 2.1 (1.2) | 4.4 (2.2) | <0.001 |
| BASMI score, median (IQR)  2-point system  10-point system  BASMI measurements, mean (SD)  - Cervical rotation  - Intermalleolar distance  - Tragus to wall  - Lateral side flexion  - Lumber Modified Schober test | 1 (1-2)  2 (1.4-2.8)  58.5 (16.6)  106.07 (14.1)  11.5 (2.9)  13.8 (4.8)  5.3 (1.7) | 2 (2-5)  3.6 (2.4-4.6)  46.8 (18.7)  99.9 (18.4)  13.5 (5.1)  11.2 (5.6)  3.9 (1.7) | 0.038  0.002  0.009  0.131  0.046  0.048  0.001 |
| Sleep disturbance, n (%) | 13 (35.1) | 18 (62.0) | 0.030 |
| HADS-A score*   - < 8 - 8-10 - ≥ 11 | 29 (78.4)  7 (18.9)  1 (2.7) | 19 (65.6)  7 (24.1)  3 (10.3) | 0.344 |
| HADS-D score°   - < 8 - 8-10 - ≥ 11 | 33 (89.2)  3 (8.1)  1 (2.7) | 18 (62.8)  6 (20.7)  5 (17.2) | 0.026 |

*HADS-A < 8 is normal,8-10 is suspicious anxiety, ≥11 is anxiety

° HADS-D < 8 is normal,8-10 is suspicious depression, ≥11 is depression
